# Bacteriological quality of raw milk marketed in and around Guwahati city, Assam, India

**DOI:** 10.14202/vetworld.2021.656-660

**Published:** 2021-03-18

**Authors:** Smita Kakati, Archana Talukdar, Razibuddin Ahmed Hazarika, Masuk Raquib, Saurabh Kumar Laskar, Girindra Kumar Saikia, Zakir Hussein

**Affiliations:** 1 Department of Veterinary Public Health, College of Veterinary Science, Assam Agricultural University, Khanapara, Guwahati, Assam, India; 2 Department of Livestock Products Technology, College of Veterinary Science, Assam Agricultural University, Khanapara, Guwahati, Assam, India; 3 Department of Microbiology, College of Veterinary Science, Assam Agricultural University, Khanapara, Guwahati, Assam, India; 4 Department of Livestock Production and Management, College of Veterinary Science, Assam Agricultural University, Khanapara, Guwahati, Assam, India

**Keywords:** coliform count, methylene blue reduction test, raw milk, total viable count

## Abstract

**Background and Aim:**

Milk is a highly perishable commodity, which is subjected to various types of contamination right from the farm level to the consumers’ table. This study aimed to assess the quality of raw milk sold in and around Guwahati city based on the microbial load.

**Materials and Methods:**

A total of 200 raw pooled milk samples collected from 25 different locations in and around Guwahati city were subjected to quality evaluation based on the methylene blue reduction test (MBRT), standard plate count, and coliform count as per the standard procedure.

**Results:**

Out of the 200 samples evaluated, more than 50% of them were graded as poor to very poor quality based on the MBRT results. None of the samples could be graded as excellent quality and only 14.5% were graded as good quality. The standard plate count and coliform count of all the raw milk samples were found to be significantly higher than the legal standard. A highly significant (p<0.01) difference was observed for standard plate count and coliform count among the different locations in and around Guwahati city.

**Conclusion:**

From the present study, it could be inferred that raw milk sold in most parts of Guwahati city do not confer to the legal microbiological standard and may pose a high risk of milk-borne illness among consumers of the city, which needs a systematic series of actions to be implemented properly.

## Introduction

Milk, in its natural form, is a unique food that provides nourishment to human beings since ages. It provides immunogenic protection and supplies nutrients, such as proteins, fat, carbohydrates, vitamins, and minerals, in an easily digestible form than any other single food [1]. There has been an increase in milk consumption in the developing countries, including India and the trend is likely to continue as the demand for animal-source food has shown a rising trend due to population growth, changing lifestyles, increasing wealth, and increasing dependence of large vegetarian population on dairy products for proteins and other micronutrients [2,3].

In recent times, there is an apparently growing concern regarding the safety and health and nutritional benefits of milk and dairy products among both urban and rural consumers. Milk being a perishable commodity is highly vulnerable to bacterial contamination as it provides a favorable medium for its growth. Today’s consumers need clean, wholesome, and nutritious food that is produced and processed in a sound, sanitary condition and are free from pathogens. Milk passes through different handlers from the farm to the table of consumers and failure to maintain cold chain may result in poor quality of milk. Raw, poorly processed and inefficiently handled milk and milk products can lead to severe food-borne illness in humans [4]. Particularly in developing countries, the shelf-life of milk and milk products tends to be shorter as the production of milk and its processing into different milk products takes place under unsanitary conditions and poor production practices [5]. Coliforms are invariably found in raw milk but with good manufacturing practices, their number can be kept very low [6]. Their presence is an indication of unsanitary production practices and/or improper handling of either milk or milk utensils [7]. Even pasteurized milk may have a high bacterial load which may be attributed to defective pasteurization machinery, microbes surviving in pasteurization, and post-pasteurized contamination due to poor processing and handling conditions and/or poor hygienic practices followed by the workers [8].

Guwahati, the capital city of Assam (24°44’ to 27°45’N latitude; 89°41’ to 96°2’E longitude), India, is the gateway of Northeast India. There is a good demand for quality milk and safe milk products in and around the city. However, limited work has been undertaken so far on the assessment of microbial load in raw milk. Considering the importance of safe food to consumers, the present study was envisaged to determine the microbial load in raw milk marketed in and around Guwahati city.

## Materials and Methods

### Ethical approval

The research was approved by the Institutional Animal Ethics Committee of Faculty of Veterinary Science, Assam Agricultural University (AAU), Khanapara (770/ac/CPCSEA/FVSc/AAU/IAEC/15-16/352) dated 10.04.2015.

### Study location and period

The work was carried out in the Department of Veterinary Public Health, Department of Livestock Products Technology, and Department of Veterinary Microbiology, College of Veterinary Science, AAU, Khanapara, Guwahati, India, from June 2015 to May 2016.

### Sampling

Two hundred raw pooled milk samples (approximately 200 mL) were collected aseptically in sterile containers from bulk milk cans from 25 different locations and markets in and around Guwahati city, as mentioned in Table-1. The samples were transported immediately to the laboratory, maintaining the cold chain and thereafter subjected to bacteriological examination within 3-4 h of collection.

**Table-1:** Different collection points of milk samples in and around Guwahati city.

Place of collection	Number of samples	Place of collection	Number of samples	Place of collection	Number of samples
Bamunimaidan	08	Jorabat	08	Panjabari	08
Basistha	08	Kahilipara	08	Satgaon	08
Bonda	08	Kalapani	08	Six Mile	08
Chandmari	08	Khanapara	08	Ten Mile	08
Eight Mile	08	Maligaon	08	Ulubari	08
Ganeshguri	08	Mathghoria	08	Bhetapara	08
Hatigaon	08	Nine Mile	08	Zoo Road Tiniali	08
Jalukbari	08	Noonmati	08		
Jonali	08	Panikhaiti	08		

### Methylene blue reduction test (MBRT)

The MBRT was carried out as per the standard method [9]. One milliliter of methylene blue (1:2500) was added to 10 mL of milk in a sterilized test tube followed by sealing it with a rubber stopper and slowly inverted it 3 times to mix the content thoroughly. The tube was then placed in a water bath maintained at 35°C and examined at regular intervals up to 8 h. Time taken for the sample to decolorize, that is, reduction time, was noted based on the time taken by dye from a definite color (blue) to colorless form (white), and the result was interpreted. The quality of milk was graded as excellent (no decolorization within 8 h), good (decolorization between 5 to and 8 h), fair (decolorization between 2 to and 5 h), poor (decolorization between 20 min and 2 h), and very poor (decolorization in <20 min), respectively.

### Total viable count (TVC) and coliform count

The TVC of the milk samples was performed by the pour plate technique [10]. Serial 10-fold dilutions were done in a sterilized normal saline solution and appropriate dilution was transferred into the sterilized Petri dishes in duplicates. Twenty milliliters of pre-sterilized, molten, and cooled plate count agar medium for TVC and violet red bile agar medium for coliform count were poured into the respective Petri dishes. The inoculum was mixed thoroughly with the media by rotating the plates several times in clockwise and anti-clockwise directions. After solidification, the plates were incubated at 37°C and 35°C, respectively, for 24-48 h. Colonies grown in respective media were counted and the counts were expressed as log_10_ cfu/mL of sample. Coliform organisms were confirmed based on the colony characteristics and production of gas in Brilliant Green Lactose Broth (BGLB) incubated at 35°C for 24-48 h.

### Statistical analysis

The data of the present study were statistically analyzed using SPSS version-20.0 software (IBM Corp., Armonk, NY, USA). One-way analysis of variance was performed for different parameters and means were compared by Duncan’s multiple range test with 1% and 5% significance level.

## Results and Discussion

### MBRT

**Figure-1:**
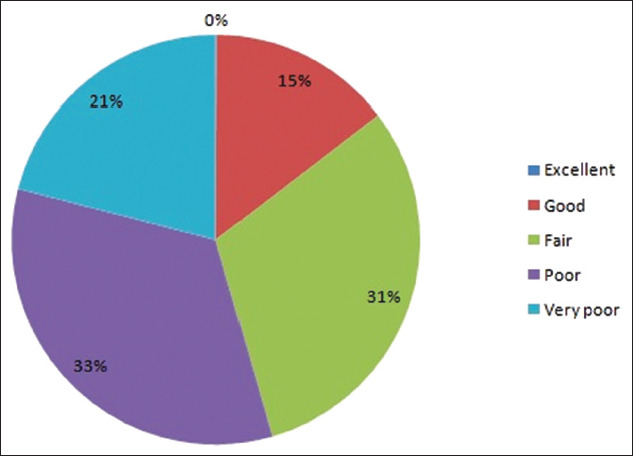
Percentage contribution of different grades of milk samples in methylene blue reduction test.

The results of MBRT of the raw milk samples collected from 25 different locations in and around Guwahati city are presented in Figure-1. None of the samples could be graded as excellent quality, while, based on the time required to reduce the dye to colorless form, only 14.5% could reduce the dye between 5 and 8 h and were graded as good quality. Out of the 200 pooled milk samples, 31% of the samples could reduce the dye between 2 and 5 h and graded as fair, 33.5% as poor (dye reduction time 20 min-2 h), followed by 21% as very poor quality (dye reduction time <20 min), respectively, irrespective of their locations with the highest percentage graded as poor quality. It could be assessed from the present study that most of the milk sold in and around Guwahati might have a poor shelf-life unless adequate intervention measures for processing are adopted at the earliest. The variation in the grading of milk samples collected from 25 different locations might be due to location specificity, seasonal variation, unhygienic production practices, inadequate maintenance of cold chain, milk composition, and initial contamination [11-15].

### Bacteriological analysis

#### TVC

The details of the TVC of raw milk samples collected from 25 different locations in and around Guwahati city are presented in Table-2. Analysis of variance revealed a highly significant difference (p<0.01) among the different locations in and around the city. From the results, it could be inferred that the microbial load in 8 mile area (14.62±0.25 log_10_ cfu/mL) was found to be significantly higher (p<0.01) compared to the samples collected from other locations, thus indicating a high level of microbial contamination. On the contrary, the lowest microbial load was observed in raw milk samples collected from Maligaon area (10.59±0.07 log_10_ cfu/mL). However, it did not differ significantly from Bamunimaidan, Panjabari, Hatigaon, Ganeshguri, and Satgaon area, respectively, while for the rest of the locations, the TVC was found to be highly significant (p<0.01). This might probably be due to high count in the raw milk due to contamination, high ambient temperature, unable to maintain cold chain during transportation, and poor and unhygienic milking practices. The samples collected from Jorabat area were found to have significantly lower microbial load (p<0.01) than 8 mile area and could be adjusted as the second most contaminated milk sold in that particular area. In the present investigation, the TVC of milk samples in all the locations was quite higher than the maximum permissible limit as suggested by the European Union, which has been reported to be 4 log_10_ cfu/mL. In the present study, the standard plate count of raw milk samples collected in and around Guwahati was found to be higher as compared to the reports of numerous workers [16-20]. However, a low bacterial count (6.32 log_10_ cfu/mL) was also reported [21] in raw milk in Guwahati as compared to the present study. Since the vendors who sell milk do not maintain the cold chains, there is every possibility for the microorganisms to grow and multiply in the milk as it provides a conducive environment, such as neutral pH, high water activity, and excellent growth medium. Therefore, it can be presumed that such milk sold in and around Guwahati will have a very short shelf-life and might compromise with the food safety issues.

**Table-2:** Total viable count (log_10_ cfu/mL) of raw milk samples collected from different locations.

Location	Mean±SE
Zoo Road Tiniali	11.27±0.14^jklmn^
Bamunimaidan	11.02±0.10^lmnop^
Bonda	11.19±0.10^klmno^
Maligaon	10.59±0.07^p^
Panjabari	11.002±0.06^lmnop^
Eight mile	14.62±0.25^a^
Basistha	11.36±0.10^jklm^
Hatigaon	10.82±0.07^nop^
Jalukbari	11.41±0.12^ijkl^
Jonali	11.16±0.14^klmno^
Jorabat	13.85±0.37^b^
Khanapara	12.96±0.20^cd^
Kahilipara	11.34±0.09^jklm^
Mathghoria	11.97±0.08^fgh^
Ganeshguri	10.90±0.09^mnop^
Paltanbazar	13.22±0.27^c^
Ulubari	12.05±0.23^fg^
Bhetapara	12.33±0.13^ef^
Satgaon	10.74±0.06^op^
Chandmari	12.63±0.28^de^
Panikhaiti	11.70±0.20^ghij^
Nine mile	11.52±0.14^hijk^
Six mile	13.27±0.25^c^
Kalapani	12.44±0.14^ef^
Noonmati	11.84±0.12^ghi^

Means in a column bearing same superscript do not differ significantly (p<0.01)

#### Coliform count

The mean±SE values of coliform counts of 200 raw milk samples collected from 25 different locations in and around Guwahati city are presented in Table-3. Analysis of variance revealed highly significant (p<0.01) differences in coliform count among the different locations in and around Guwahati city. The highest count was recorded in both 6 mile and Panjabari areas (7.50±0.07 log_10_ cfu/mL) and was found to be highly significant from other locations, except Zoo Road Tiniali area (7.32±0.10 log_10_ cfu/mL) which was numerically lower. Lowest coliform count was recorded in Maligaon area (6.34±0.046 log_10_ cfu/mL) but did not differ significantly from Basistha (6.46±0.05 log_10_ cfu/mL), Kahilipara (6.49±0.05 log_10_ cfu/mL), Ganeshguri (6.49±0.06 log_10_ cfu/mL), Paltanbazar (6.50±0.05 log_10_ cfu/mL), Bhetapara (6.50±0.05 log_10_ cfu/mL), Satgaon (6.44±0.05 log_10_ cfu/mL), and Kalapani (6.39±0.04 log_10_ cfu/mL) area although the coliform counts were numerically higher than Maligaon area. All the samples were found to be positive for gas production on inoculation in BGLB medium. The coliform count of the raw milk samples recorded in the present investigation was significantly higher than the maximum permissible standard. The present findings are in close agreement with the findings of an earlier report [22]. On the contrary, the coliform counts in the present investigation were significantly higher than some of the other reports [23-28]. This indicates selling of unhygienic milk in and around Guwahati. This might have attributed to the current observations on dirty udder conditions, unhygienic milking procedures and housing environment, contamination of milk with dung, dirty utensils, and improper drainage system maintained in the respective dairy farms, which need prioritizing interventions influencing the structure and development of unorganized private dairy sector.

**Table-3:** Coliform count (log_10_ cfu/mL) of raw milk samples collected from different locations.

Locations	Mean±SE
Zoo Road Tiniali	7.32±0.10^ab^
Bamunimaidan	6.59±0.05^ghi^
Bonda	6.77±0.10^fg^
Maligaon	6.34±0.05^j^
Panjabari	7.50±0.07^a^
Eight mile	7.08±0.08^cd^
Basistha	6.46±0.05^ij^
Hatigaon	7.04±0.08^cd^
Jalukbari	6.62±0.09^fghi^
Jonali	6.69±0.08^fgh^
Jorabat	7.13±0.09^bcd^
Khanapara	7.03±0.08^cd^
Kahilipara	6.49±0.05^hij^
Mathghoria	7.21±0.09^bc^
Ganeshguri	6.49±0.06^ij^
Paltanbazar	6.50±0.05^hij^
Ulubari	6.59±0.06^ghi^
Bhetapara	6.50±0.05^hij^
Satgaon	6.44±0.05^ij^
Chandmari	7.11±0.10^cd^
Panikhaiti	7.07±0.09^cd^
Nine mile	6.97±0.08^de^
Six mile	7.50±0.07^a^
Kalapani	6.39±0.04^j^
Noonmati	6.82±0.08^ef^

Means in a column bearing same superscript do not differ significantly (p<0.01)

## Conclusion

Based on the findings of the present investigation, it could be concluded that there is a high risk of milk-borne illness among consumers due to inadequate farm-to-table practices. There is an utmost need to have a systematic awareness program with a follow-up mechanism to educate the dairy farmers on hygienic production practices at farm level to produce clean and wholesome milk as well as the retailers to promote maintenance of cold chain during the entire selling process.

## Authors’ Contributions

RAH, AT, and MR conceptualized and designed the work. SK carried out the experimentation. AT, RAH, MR, SKL, GKS, and ZH provided the necessary guidelines. SK, AT, RAH, and MR drafted the manuscript and all authors contributed critically to revise the manuscript. All authors have read and approved the final manuscript.
